# ALV Integration-Associated Hypomethylation at the TERT Promoter Locus

**DOI:** 10.3390/v10020074

**Published:** 2018-02-10

**Authors:** Gary Lam, Karen Beemon

**Affiliations:** Department of Biology, Johns Hopkins University, Baltimore MD 21218, USA; glam5@jhu.edu

**Keywords:** ALV, TERT, DNA methylation, cancer, viral integration

## Abstract

Avian leukosis virus (ALV) is a simple retrovirus that can induce B-cell lymphoma in chicken(s) and other birds by insertional mutagenesis. The promoter region of telomerase reverse transcriptase (TERT) has been identified as an important integration site for tumorigenesis. Tumors with TERT promoter integrations are associated with increased TERT expression. The mechanism of this activation is still under investigation. We asked whether insertion of proviral DNA perturbs the epigenome of the integration site and, subsequently, impacts the regulation of neighboring genes. DNA cytosine methylation, which generally acts to suppress transcription, is one major form of epigenetic regulation. In this study, we examine allele-specific methylation patterns of genomic DNA from chicken tumors by bisulfite sequencing. We observed that alleles with TERT promoter integrations are associated with decreased methylation in the host genome near the site of integration. Our observations suggest that insertion of ALV in the TERT promoter region may induce expression of TERT through inhibition of maintenance methylation in the TERT promoter region.

## 1. Introduction

Avian leukosis virus (ALV) is a simple retrovirus that can induce B-cell lymphoma in chickens and other fowl by means of insertional mutagenesis. Proviral integration can upregulate the expression of proximal genes through enhancer and promoter elements in the viral long terminal repeat (LTR) sequences [1,2]. Previous studies have shown common integration sites in ALV-induced lymphomas near *MYC*, *MYB*, *BIC* and, more recently, the telomerase reverse transcriptase (TERT) genes [3,4,5,6,7,8,9]. Previously, we found that integrations in the TERT promoter region were one of the most clonally expanded—or most abundant unique—integrations in tumors tested from ALV infected chickens [3,9]. This suggests that TERT promoter integrations occurred early during ALV-infection in tumors with abundant copies of a unique TERT integration, implicating them as important early events in tumorigenesis [3,9].

TERT encodes the catalytic subunit of telomerase, which has been shown to be upregulated in 90% of different types of human cancers surveyed, including lymphomas [10]. Elevated TERT expression contributes to telomerase-dependent maintenance of telomeres that is often required for long-term proliferation and survival of cancer cells [11]. Similar phenotypes can be achieved through a telomerase-independent process, known as alternative lengthening of telomeres (ALT), which has been observed in both humans [12,13] and chickens [14]. Expression of TERT is tightly regulated through many mechanisms, including epigenetic modification of the promoter region to regulate telomerase activity in most somatic cells [15,16]. Systematic analysis of the Cancer Genome Atlas database revealed that methylation of the TERT promoter region is one of the most prevalent markers associated with TERT expression in human cancers, in addition to the discovery of common somatic point mutations in the TERT promoter [17,18,19].

DNA methylation is generally associated with repression of gene expression and occurs almost exclusively at regions of DNA where a cytosine nucleotide is followed by a guanine nucleotide (CpGs) in vertebrates [20,21,22]. The vast majority of DNA is highly methylated at CpGs; however, a small fraction of DNA comprising CpG islands, areas containing a high concentration of CpGs (at least 200 bp long with >60% GC), show differential methylation during development and disease states [20,21,22]. These CpG islands are frequently associated with gene promoters [20,21,22]. In the case of TERT, the relationship between TERT promoter methylation and expression has proven to be complex and is still under active investigation. Surprisingly, early studies suggest a direct relationship between TERT promoter methylation and expression, and, subsequently, telomerase activity [23,24,25,26,27]. In multiple studies, normal human somatic cells that do not express TERT are associated with unmethylated or hypomethylated promoters, while some cancer lines with completely hypermethylated TERT promoter regions express TERT [23,24,25,26,27]. In contrast, other reports of TERT promoter DNA methylation suggest that methylation is associated with gene silencing [28,29,30]. Further investigations reveal that the activation of TERT expression can be allele-specific in cancer cells, which are under pressure to maintain active alleles protected from DNA methylation [31]. Most recently, common TERT promoter mutations are shown to be associated with allele-specific hypomethylation of the TERT promoter in cancer cells with TERT expression [32].

DNA methylation also plays an important role in the regulation of retroviral proviruses. First introduced by Katz and co-workers, evidence of proviral DNA methylation was observed in a rat restriction cell line (XC) that was established from rat sarcoma tumors induced through heterotransplantation by inoculating newborn rats with suspensions of Rous sarcoma tissue [33,34]. Using this model, Svoboda and co-workers demonstrated that DNA methylation was involved in transcriptional silencing of avian proviruses [35,36]. Daxx, a cytoplasmic Fas death domain-associated protein, was later discovered to be required for long-term maintenance of silencing and full viral DNA methylation of avian proviruses in human cells [37].

Further investigation revealed a dynamic relationship between the methylation state of the proviruses and the context of the integration site. The integrations of ALV-related retroviruses like Rous sarcoma virus (RSV) and Moloney murine leukemia virus (MLV) can perturb the methylation state of flanking host DNA in different ways. RSV integration has been associated with transient hypomethylation of flanking genomic DNA in hamster cells [38]. In contrast, MLV integration is associated with de novo methylation in mice [39]. Proviruses integrated close to transcriptional start sites of active genes may have long-term transcriptional activity and be resistant to transcriptional silencing by DNA methylation; in contrast, proviruses in intergenic regions tend to become transcriptionally silenced [40]. Studies with other human viruses suggest that there is a direct correlation between the methylation state at the site of integration prior to proviral integration and the methylation status of the provirus [41,42]. Ultimately, local changes in DNA methylation are dependent on a growing list of factors that include the host cell properties, integrating virus, and the site of integration.

**Figure 1:**
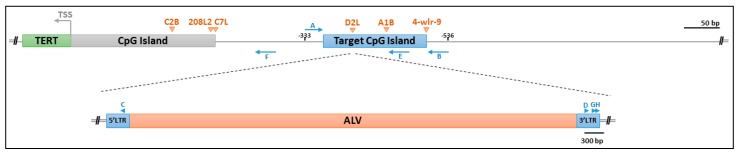
Schematic illustration of PCR primers, the cytosine-guanine dinucleotide (CpG) islands, and avian leukosis virus (ALV) telomerase reverse transcriptase (TERT) integrations in the chicken TERT promoter region. Primers are labeled A–H and depicted as letters and blue arrows. The targeted CpG island of interest is depicted within 333–536 bp upstream of the TERT transcriptional start site (TSS). The six ALV integrations tested are labeled and indicated with orange arrows at the site of integration. At the bottom, a schematic of ALV provirus in tumor D2L is depicted with primers labeled in the long terminal repeats (LTRs).

Chicken and human telomerase activity and telomere biology share some key similarities. High levels of telomerase activity are observed in early stage chicken embryos and human prenatal organs, and telomerase activity is downregulated in a temporal and tissue-specific manner for most somatic tissues [43,44,45]. With some exceptions, constitutive activity is observed in renewable tissue types and diminished telomerase activity in most differentiated somatic tissues [43,44,45]. Division-dependent telomere shortening occurs in chicken somatic tissues, and telomerase activity upregulation is present in transformed chicken cells [46]. Furthermore, chicken TERT also has CpG islands spanning from the TERT promoter region and into the coding region. More specifically, there is a smaller CpG island from −337 to −471 relative to the TERT transcriptional start site (TSS), and a larger one from −200 to +746 spanning across the first TERT exon and into the first intron (Figure 1). In comparison, human TERT has CpG islands spanning from −846 to +1178 relative to the TERT TSS.

The influence of ALV integration on the methylation of the chicken TERT promoter has not been characterized previously in ALV-induced lymphomas. Our lab has observed that the clonally expanded integrations in the TERT promoter remain transcriptionally active in chicken tumors. Expression analysis revealed the upregulation of a novel TERT antisense promoter-associated (TAPAS) long non-coding RNA (lncRNA), in chicken tumors with TERT promoter integrations. TAPAS transcripts corresponded to a region downstream of the common ALV integration site in the TERT promoter region. Expression of TAPAS was shown to be partly driven by the ALV proviral promoter, which was supported by the detection of spliced proviral transcripts fused with TAPAS [8]. Furthermore, the same subset of tumors was associated with increased TERT expression [7,8]. Thus, we studied the epigenetic state of the ALV provirus and the TERT promoter in chicken lymphomas with clonally expanded TERT promoter integrations. To better understand the interplay between the epigenome of the host DNA and provirus, we compared the DNA methylation status of the unoccupied allele (without a TERT promoter integration) with the occupied allele (with a TERT promoter integration) in individual tumors with known clonally expanded TERT integrations. In this study, we found that ALV integrations in the TERT promoter are associated with hypomethylation of the flanking host genomic DNA and of the ALV LTRs.

## 2. Materials and Methods

### 2.1. Tumor Induction and Samples

Groups of 5- and 10-day-old chicken embryos were infected with ALV by injection with either ALV-LR9, ALV-ΔLR9, ALV-G919A, or ALV-U916A as described previously [3,47]. Chickens injected include inbred single comb (SC) White Leghorn line embryos from Hy-Line International (Dallas Center, IA, USA) and specific pathogen free embryos from Charles River (North Franklin, CT, USA). ALV-LR-9 mutants have a deletion (ALV-ΔLR9) or silent mutation (ALV-G919A and ALV-U916A) in a 5′ regulatory element that results in rapid onset of B-cell lymphomas in chickens. Chickens were observed daily after hatching and euthanized when moribund or at the end of 10–12 weeks. Once sacrificed, tumor tissue and normal tissue were harvested from infected birds and frozen at −80 °C. Normal tissues were collected from uninfected chickens that were sham injected with media as controls. In total, five normal tissues (bursa, kidney, liver, spleen and non-tumor tissue from an infected bird, C2K), nine tumors without any known TERT promoter integrations, nine tumors with known TERT promoter integrations, and two chicken cell cultures were used for bisulfite sequencing (Table 1) [3,9].

Chicken experiments were performed at the University of Delaware. These experiments were approved by the Institutional Animal Care and Use Committee (AUP Number 1271-2016-2), which was renewed on 1 August 2016.

### 2.2. DNA Extraction and Bisulfite Treatment

50 mg of tissue was homogenized with a Kimble-Chase Kontes pellet pestle and digested with proteinase K at 50 °C for 15 h. DNA was extracted with two rounds of phenol-chloroform extraction, with a 2 µg RNase A treatment for 1 h at 37 °C in between the rounds, followed by ethanol precipitation. DNA concentration was measured with a Thermo Scientific Nanodrop 2000c instrument (Waltham, MA, USA). For bisulfite treatment, an optimized protocol was adapted from Pappas et al. [48]. In brief, 5 µg of genomic DNA was sonicated briefly with a Bioruptor UCD-200 instrument (Diagenode Inc., Denville, NJ, USA). DNA samples were then denatured using 3 N NaOH at 37 °C for 15 min. Sulfonation of unmethylated cytosines was performed by the addition of sodium bisulfite-hydroquinone solution in a thermal cycler at 95 °C for 4 min followed by 55 °C for 2 h. DNA was then desalted using a Thermal Scientific GeneJet PCR purification Kit (Waltham, MA, USA) and deaminated with the addition of 3 N NaOH at 37 °C for 15 min. Lastly, desulfonation and precipitation were performed by adding glycogen, 10 M ammonium acetate, chilled in 95% ethanol and incubated overnight at −20 °C. Samples were centrifuged at 13,000× *g* for 30 min, washed with 70% ethanol, air-dried, and stored at −20 °C for PCR amplification and sequencing.

### 2.3. PCR Amplification and Sequencing Analysis

Target sequences for tumor samples 4-wlr-9, A1B, and D2L were amplified by one round of PCR using bisulfite treated DNA. A schematic representation of the positions of corresponding primers, targeted region, and ALV integration sites are shown in Figure 1. Henceforth, alleles without a TERT promoter integration will be referred to as unoccupied alleles and alleles with an integration as occupied alleles. For amplification of the unoccupied alleles, a target CpG island within 333–536 bp upstream of the chicken TERT TSS was amplified using primer A (GTTTTTGTTTTGTTTTTGAG-GAGAT) and primer B (AACTAAAAATTTTCTCTATCAAATTATATT). For the amplification of occupied alleles, primers flanking the proviral-host junction at the site of integration were used. For 4-wlr-9 and A1B, primer A and primer C (CAACCCAAATACACACCAATATAATAA) were used to amplify the 5′LTR-host junction. For D2L, primer B and primer D were used to amplify the 3′LTR-host junction. Amplification of occupied alleles in C7L, C2B, and 208L2 required a semi-nested PCR approach. The first round of PCR was performed using primer E (TCACAAAAATAAATAAAAAA-CATTACTT) and primer G (TATATTGGTGTGTATTTGGGTTGAT). In the second round, 3′LTR was amplified using primer F (AAAAAAACCTCTAAAATCACTTAATCC) and primer G, while the target CpG island was amplified using primer H (ATGTAGAAGTAGAAGGTT-TTATTT) and primer F. PCR amplifications were performed using Thermo Scientific Phusion U Hot Start DNA polymerase (Waltham, MA, USA) with optimized conditions adapted from manufacturer’s instructions.

PCR amplicons were analyzed by conventional sequencing provided by Eurofins Genomics services (Louisville, KY, USA). Quantification of methylation was performed manually by digitally measuring the peak height of chromatograms. For each CpG site, percent methylation was calculated by dividing the cytosine peak height by the total peak height of cytosine and thymidine and multiplying by 100. Percent methylation means were calculated by averaging percent methylation of all CpG sites sequenced region of one representative sample. All unmethylated CpG sites had undetectable cytosine, or showed only thymidine peaks. All non-CpG cytosine showed complete conversion to thymidine. At least two technical repeats were performed from extracted genomic DNA.

PCR amplification and sequencing analysis of the larger CpG that was closer to the TSS proved to be beyond the limitations of the method described and, subsequently, was not analyzed in this study.

## 3. Results

### 3.1. Chicken TERT Promoters Were Significantly Methylated in Unoccupied Alleles

**Figure 2:**
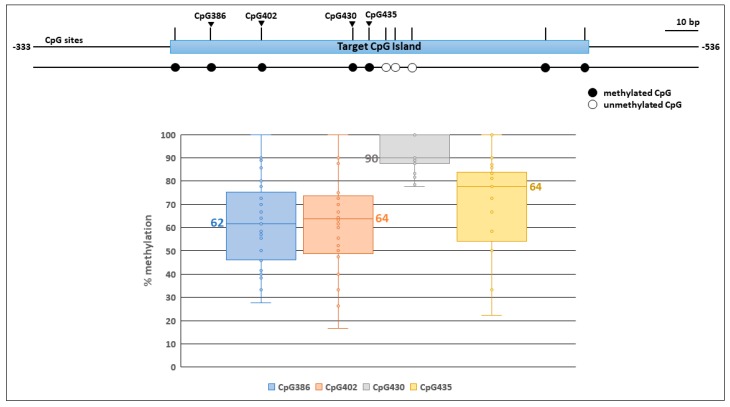
CpG methylation profiles of the targeted TERT promoter region in unoccupied alleles. Schematic representation of the CpG dinucleotide distribution is shown at the top. Four CpG sites of interest (CpG386, CpG402, CpG430, and CpG435) are indicated. Bisulfite sequencing analysis of the target region is depicted as a linear array of open circles representing non-modified CpG residues and closed circles representing methylated CpG residues. At the bottom, a box and whisker plot shows percent methylation distribution between each sample tested at four CpG sites that varied in the targeted CpG island of the TERT promoter region. Each dot may represent more than one measure of percent methylation in one sample at the indicated CpG site when percent methylation is the same. A total 25 samples are tested across the four CpG sites. Median percent methylation for each site is indicated next to each box in the corresponding color.

Limited data were available about the methylome of chicken tumors. In order to assess the effects of ALV integration on the methylation status of the TERT promoter, we required data on the methylation status of a CpG rich region in the TERT promoter of unoccupied alleles. We performed conventional bisulfite sequencing of genomic DNA samples that include five normal tissues, two chicken cell culture samples, nine tumors that do not have clonal TERT promoter integrations, and nine tumors that do have clonal TERT promoter integrations (Table 1). Sequencing was performed on PCR amplicons 333–536 bp upstream of the chicken TERT TSS of the unoccupied allele (Figure 1). This specific region was selected because multiple chicken tumors have neighboring TERT promoter integrations (Figure 1). CpG site distribution is depicted in this region in Figure 2. All tissues tested show the same pattern of DNA methylation across the target CpG island (Figure 2).

Variation in methylation between samples was observed at 4 of 10 CpG sites measured: CpG386, CpG402, CpG430, and CpG435 (Figure 2). Percent methylation at CpG386, CpG402, CpG430, and CpG435 ranged from 28–100, 17–100, 78–100, and 22–100% with a median of 62, 64, 90, and 64%, respectively (Figure 2). The remaining CpG sites were either unmethylated or completely methylated as indicated, across all samples tested (Figure 2). Variation in methylation between the samples did not separate into any obvious groups, like normal and tumor tissues. Taken together, significant methylation of unoccupied alleles was observed in both normal and tumor tissues with no obvious trends between sample groups.

### 3.2. ALV LTRs of Proviruses in the TERT Promoter Were Not Methylated

**Figure 3:**
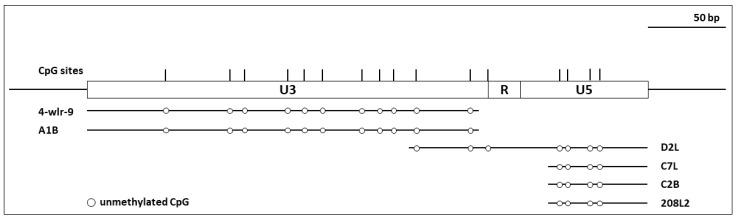
CpG methylation profiles of TERT promoter proviral LTRs in selected tumor samples. Schematic representation of the CpG dinucleotide distribution in the ALV LTR is shown at the top. Bisulfite sequencing analysis of the LTR sequences are depicted as a linear array of open circles representing non-modified CpG residues and closed circles representing methylated CpG residues. Each line represents a representative sequenced result of the LTR region from host-proviral PCR amplicons. No methylation was detected in proviruses of tumor tissues tested.

To examine the methylation state of ALV LTRs, we performed conventional bisulfite sequencing of six different tumors (4-wlr-9, A1B, D2L, C7L, C2B, and 208L2) with known clonally expanded TERT promoter integration sites. Sequencing was performed on PCR amplicons generated using primers flanking the proviral-host junction (Figure 1). With ALV integrations downstream of the target CpG island, tumors 4-wlr-9 and A1B were used to analyze the U3 region of the 5′LTR. In both cases, there were no detectable evidence of modified CpG dinucleotides. With integrations upstream of the target CpG island, tumors D2L, C7L, C2B, and 208L2 were used to analyze the R and U5 region of the 3′LTR. No evidence of methylation was detected in the 6 samples. Taken together, there was no evidence of methylated CpG dinucleotides throughout the LTR (Figure 3). Thus, ALV integration into the chicken TERT promoter region is not associated with de novo methylation of LTRs in B-cell lymphomas.

### 3.3. Occupied Alleles in the TERT Promoter had Decreased Methylation in Flanking Host DNA

To examine the effects of ALV integration on the methylation of the flanking host DNA in the TERT promoter, bisulfite sequencing was performed on six different tumors (4-wlr-9, A1B, D2L, C7L, C2B, and 208L2). They all had known clonally expanded TERT promoter integrations near the target CpG island. For each tumor, two parallel sequencing experiments were performed. For the unoccupied alleles, PCR amplicons were generated from the region that is 333–536 bp upstream of the chicken TERT TSS containing the target CpG island, using primers for the host genome. For the occupied alleles, PCR amplicons were generated using an LTR specific primer for the LTR closest to the target CpG island of the TERT promoter and a shared primer in the host genome (Figure 1).

**Figure 4:**
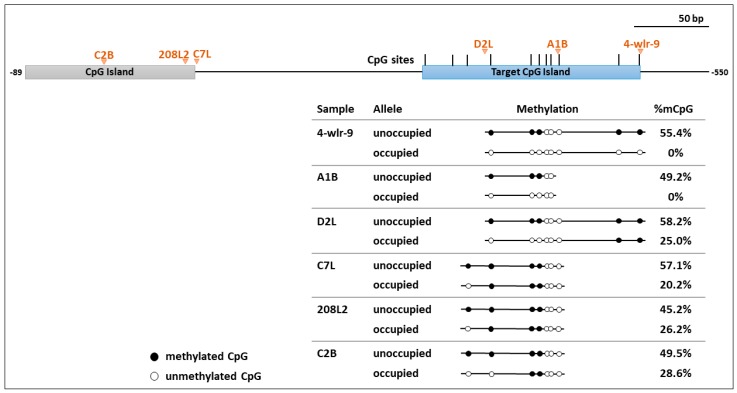
CpG methylation profiles of host genomic DNA in selected tumor samples. Schematic representation of the CpG dinucleotide distribution in the target CpG island in the TERT promoter region is shown at the top. The six TERT promoter integrations tested are labeled and indicated with orange arrows at the site of integration. For each tumor sample listed, bisulfite sequencing analyses are depicted as linear arrays of open circles representing non-modified CpG residues and closed circles representing methylated CpG residues. For each tumor sample, CpG methylation data in the target CpG island is shown for the unoccupied allele and the occupied allele. Corresponding mean percent CpG are listed, which were calculated by averaging percent methylation of all CpG sites in the displayed array.

In all six cases, a decrease in methylation was detected in the occupied allele compared to the paired unoccupied allele (Figure 4). Mean percent methylation decrease ranged from a complete loss of methylation in 4-wlr-9 and A1B to an approximately two-fold decrease in D2L, C7L, C2B, and 208L2 (Figure 4). Decreases in methylation at individual CpG sites appeared to be more dramatic closer to the site of integration (Figure 4). For example, occupied alleles of D2L show a complete loss of methylation at CpG sites immediately downstream of the integration but show retention of methylation at CpG sites farther downstream. Being farther upstream, occupied alleles from C2B, 208L2, and C7L show methylation at some sites that are unmethylated in occupied alleles of D2L, A1B, and 4-wlr-9. Taken together, ALV integrations near methylated sites in the host genome are associated with a decrease in methylation of the local host genome.

## 4. Discussion

Our lab has previously shown that the TERT promoter region is a common integration site in ALV-induced B-cell lymphoma. These integrations are the most clonally expanded in a subset of tumors, suggesting that proviral integration into this region is an important early event in lymphoma development [3,9]. Tumors with TERT promoter integrations are associated with increased TERT expression [7,8]. The mechanism of this activation is presently under investigation. The present study investigates the influence of ALV integration on the TERT promoter region methylation status at the site of integration in ALV-induced lymphoma. In this study, we observed that the chicken TERT promoter is significantly methylated in unoccupied alleles across normal and cancer tissues. Tested tumors with TERT promoter integrations appear to have proviruses that are free of any detectable CpG methylation in the LTRs. Adjacent host sequences show a decrease in methylation in occupied alleles compared to unoccupied alleles.

DNA methylation of promoter regions is often associated with transcriptional silencing [20,21,22]. We observed significant methylation in the chicken TERT promoter region of unoccupied alleles, suggesting that in normal tissues and tumors, cellular factors were actively repressing transcription of TERT by means of DNA methylation. This is consistent with the downregulation of telomerase activity observed in chicken somatic tissues after the embryonic stage [43,44,45,46]. In contrast, there was a complete lack of detectable methylation in the ALV LTRs of proviruses in the TERT promoter region in lymphomas. As methylation in the LTR generally suppresses ALV proviral transcription [35,38,40], this result suggests that the ALV proviruses were likely transcriptionally active, which is consistent with the upregulation of spliced proviral transcripts containing TAPAS [8].

More importantly, we observe an allele-specific decrease in methylation in the host DNA near the site of ALV integration in the TERT promoter in the occupied alleles. This decrease in methylation may lead to derepression of the TERT gene and contribute to the observed increase in TERT expression in an allele-specific manner. Additional studies are required to determine if the methylation status directly impacts TERT expression. This is confounded by the presence of the LTR enhancer that has been demonstrated to activate transcription from the TERT promoter in cultured cells [7]. To decouple the effects on TERT expression from the LTR enhancer and integration site methylation, future studies comparing expression from reporter constructs comprised of methylated and unmethylated TERT promoter region and ALV LTR may provide further insight. Taken together, the pattern of methylation observed would suggest that methylation behaves in the conventional sense as a repressive marker of expression at the chicken TERT promoter.

Presently, the relationship between human TERT promoter methylation and TERT expression is still under investigation. On the one hand, increased TERT expression correlates with the hypermethylation of the TERT promoter region in many human cancers [17]. The mechanism of activation of TERT expression by increased TERT promoter methylation is presently unclear in humans. On the other hand, TERT promoter hypermethylation is associated with gene silencing [28,29,30]; thus, in the tumor environment, one would expect selection for cells that acquired different mechanisms to protect the TERT promoter from DNA methylation. Our observations support the latter. Furthermore, the allele-specific hypomethylation of TERT promoter region that is associated with ALV integration is in agreement with recent work in humans. This idea of allele-specific activation of TERT was recently explored by multiple groups in different contexts. Common somatic TERT promoter mutations were recently discovered in many human cancers [18,19]. Further investigation revealed that these somatic mutations were associated with an allele-specific switch to an active transcriptional state and monoallelic TERT expression of the allele containing the TERT promoter mutation across multiple human cancers [49,50]. More recently, allele-specific hypomethylation was associated with TERT promoter mutations [32].

In regards to the seemingly contradictory observations about TERT promoter methylation, TERT hypermethylation may represent a broadly applicable prognostic marker for TERT expression and cancer progression [51,52] that is distinct from what is observed in cancers with observed mechanisms of TERT activation like proviral- or mutation-associated hypomethylation. Presently, most of the published data looked at the average methylation across a population of alleles in tumors. Reanalyzing these samples and data, taking in account allele-specific methylation, may present novel insight into the relationship between TERT promoter methylation and cancer. Perhaps in some human cancers, there is a decrease in TERT promoter methylation in the activated allele while on average there was an increase in methylation across the population of alleles when compared to corresponding normal tissue.

Concurrently, investigations into the effects of human integrating viruses on methylation in tumors were performed. Human herpesvirus 6B has been shown to integrate into subtelomeric regions and induce hypomethylation in these regions [53]. Notably, the human TERT gene is located in the subtelomeric region of chromosome 5. Integrated human papillomavirus and hepatitis B virus show allele-specific methylation changes at the site of integration [41,42]. In these studies, methylation of the integrated viral genomes directly correlated with the methylation state of the integrated site prior to viral integration at specific sites tested [41,42]. Interestingly, the most frequent hepatitis B virus integrations were found in the TERT promoter region [54]. To our knowledge, the methylation status at these integration sites have not been tested.

Future studies into the impact of human integrating viruses on DNA methylation and TERT expression in human cancers offers an exciting direction to test our observations observed in chicken tumors. Furthermore, chickens with TERT promoter integrations represent a subset of a larger collection of ALV-induced lymphomas. Ongoing work with other common integration sites may present novel trends that may provide further insight into the role DNA methylation plays in cancer. From these initial findings, a more comprehensive approach, such as high-throughput sequencing of integration sites, may be designed to address the specificity of this phenomenon. 

## 5. Conclusions

We propose an additional selection factor in the growing model of ALV-induced tumorigenesis in chicken B-cell lymphoma. During early embryonic development, chicken embryos can become infected with ALV. Cells that acquire proviral integrations into the TERT promoter receive an advantageous enhancement to proliferation and survival by means of TERT upregulation. This upregulation is in part associated with changes in the methylation state of the TERT promoter through the insertion of ALV. Additional studies are required to understand the mechanism and impact of the methylation changes on TERT expression and the specificity of this phenomenon.

## Figures and Tables

**Table 1:** Chicken samples tested for DNA methylation.

Sample Group	Tissue Type
Normal control tissues	normal bursa (NB), normal kidney (NK), normal liver (NL), normal spleen (NS), C2K
Chicken cell culture	primary chicken embryo fibroblast (CEF), bursal lymphoma cell line (DT-40)
Tumors without telomerase reverse transcriptase (TERT) promoter integration	205L1, 209L, 214L4, 215K1, 218K, 796L, 223K, 791L1, B8L
Tumors with TERT promoter integration	206L1, 208L2, A1B, C2B, C6L, C7B, C7L, D2L, 4-w-lr-9
